# Unexpected Complication Ten Years after Initial Treatment: Long-Term Report and Fate of a Maxillary Premolar Rehabilitation

**DOI:** 10.1155/2018/3287965

**Published:** 2018-09-16

**Authors:** Davide Augusti, Gabriele Augusti

**Affiliations:** DDS, Private Dental Practice, Cosmetic and Restorative Dentistry, Via Papa Giovanni XXIII 37, 20091 Bresso Milan, Italy

## Abstract

Full-coverage restorations represent a well-known rehabilitation strategy for compromised posterior teeth; in the last years, new ceramic materials like zirconia have been introduced and widely adopted for the prosthetic management of molar and premolar areas. A long-term follow-up of a maxillary premolar rehabilitation using a veneered zirconia crown is presented; after ten years of uneventful clinical service of the tooth-restoration complex, a serious complication—namely, a vertical root fracture (VRF)—occurred. An extended time lapse (9 years) between the end of restorative procedures and development of symptoms due to VRF has been observed. On the other hand, a complete functional and esthetic integrity of the zirconia crown (without chippings or crack development) is documented along the follow-up period. Due to periodontal breakdown and severity of fracture, the premolar was extracted. The illustrations of our late failure, aetiological factors, and available data on the literature regarding VRF are addressed. Patients and clinicians should be aware of potential occurrences of some long-term, serious complications when dealing with previously treated and/or structurally weakened teeth. The development of a VRF might be unexpected and might occur many years after the end of tooth rehabilitation, despite adoption of contemporary restorative protocols and techniques.

## 1. Introduction

The prosthetic crown placement on posterior, endodontically treated teeth has been suggested in the literature to improve their long-term prognosis [1–4]. According to Aquilino and Caplan, endodontically treated teeth without a crown restoration after filling of the canals were lost at a 6.0 times greater rate than teeth with full coverage after obturation [1]. An old study investigated a large number of extracted root-canal-treated (RCT) teeth [5]: the author found significant differences in the longevity between crowned teeth and those without cuspal protection, in favor of the former (average time before extraction of 87 and 50 months, respectively). In particular, RCT teeth restored with indirect prosthetic restorations (i.e., crown, bridge, and gold partial crown, with or without prefabricated posts) demonstrated a significantly lower mean fracture rate (14-year survival before fracture) than non-vital teeth provided with just a composite filling (10-year survival before fracture) [6].

New all-ceramic prosthetic materials, like yttrium tetragonal zirconia polycrystal (Y-TZP), represent a clinical alternative to the metal frameworks of single crowns, bridges, and also for minimally invasive, adhesively luted resin-bonded fixed partial dentures [7, 8]. This material offers high resistance to masticatory stresses and effectively reproduces the appearance of a natural dentition [9].

The reported contemporary survival rate for all-ceramic, single restorations is generally high: according to Ozer et al., more than 90% of posterior porcelain fused-to-zirconia crowns survived after a mean period of 7.4 years [10]. A meta-analysis of Sailer et al. demonstrated a 96% survival rate for densely sintered zirconia fixed dental prostheses at a 5-year follow-up; a statistically similar value (94.7%) was also found for metal-ceramic restorations [11]. A recent systematic review set the success (absence of any kind of technical or biological complications) of single crowns on RCT teeth at 92%, after 6 years of service [12].

However, extensive operative procedures might be required on compromised teeth before they receive a full occlusal coverage. The long-term prognosis of multidisciplinary treated teeth (endodontic, periodontal, prerestorative, and prosthetic steps) might decrease substantially. For example, Moghaddam et al. have found a survival rate of 83% and of 51% for multidisciplinary treated teeth at 10- and 13-year recalls, respectively [13]. In other words, potential complications should be expected on severely damaged teeth when they are restored with multidisciplinary procedures and followed up for a long time.

Vertical root fractures (VRF) are defined as longitudinally oriented cracks confined to the root [14]: they are included among the potential reasons for failures of crowned teeth [15], leaving the clinician with few or limited treatment options. According to their 3-dimensional direction and extension, VRF can be classified as partial or complete [16]: a full separation and displacement of root fragments might eventually lead to tooth extraction [17, 18]. VRF are encountered more frequently on specific tooth types due to biomechanical factors: first lower molars (mesial root) and upper/lower premolars are predominantly affected, mainly due to a reduced mesio-distal diameter of the roots [19]. The reasons for extraction of a group of RCT teeth were analysed in a prospective study: the authors showed that 13.4% of the specimens were affected by a vertical root fracture [20]. An overall prevalence of 3 to 5% has been reported for that kind of complication; however, an underestimation of the problem might exist and be related to unrecognized cracks after extraction and/or other diagnostic difficulties [21].

The purpose of this paper is to report a long term, 10-year follow-up case of a maxillary premolar restored by contemporary endodontic and prosthetic procedures: after several years of uneventful service of the tooth-restoration complex, a serious complication—vertical root fracture—occurred. About 18% of VRFs are developed within 1 year from endodontic procedures [22]; in another study, the failure of teeth (i.e., extraction) associated to longitudinal fracture was established 1–5 years postoperatively [19]. In our case, an extended time lapse (9 years) between the end of restorative procedures and development of symptoms due to VRF has been observed. At the same time, a complete functional and esthetic integrity of the zirconia crown (without chippings or crack development) is documented along the entire follow-up period. After case illustration, potential causes of the failure and analysis of available data in the literature related to VRF will be addressed.

## 2. Case: Report Presentation

### 2.1. Patient Presentation and Preliminary Care

**Figure 1:**
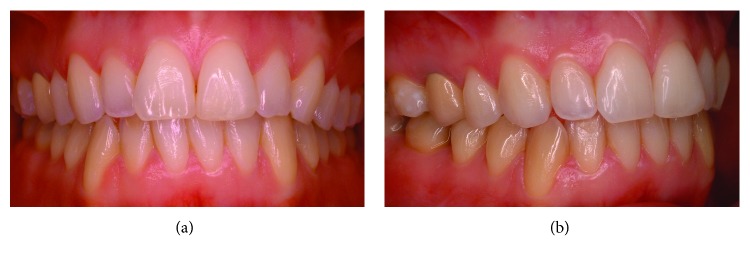
Preoperative intraoral frontal (a) and lateral (b) views showing the preexisting upper second premolar crown (tooth 1.5).

A healthy female Caucasian patient (M.M, 46 years old) with an overall good oral hygiene attitude presented at our private dental practice in 2008; following a preliminary full-mouth dental bleaching and direct conservative therapies (i.e., restorations at elements 1.6–1.7) at the right maxillary quadrant, a decision was made to replace an old metal-ceramic prosthetic crown of tooth 1.5. Lateral and occlusal views of the preexisitng restoration are shown in Figures 1(a) and 1(b). Esthetic reasons guided the replacement, in order to achieve a new optimal integration with adjacent bleached teeth. At start of the new restorative cycle, informed consent was obtained.

### 2.2. Treatment Plan for Upper Premolar

**Figure 2:**
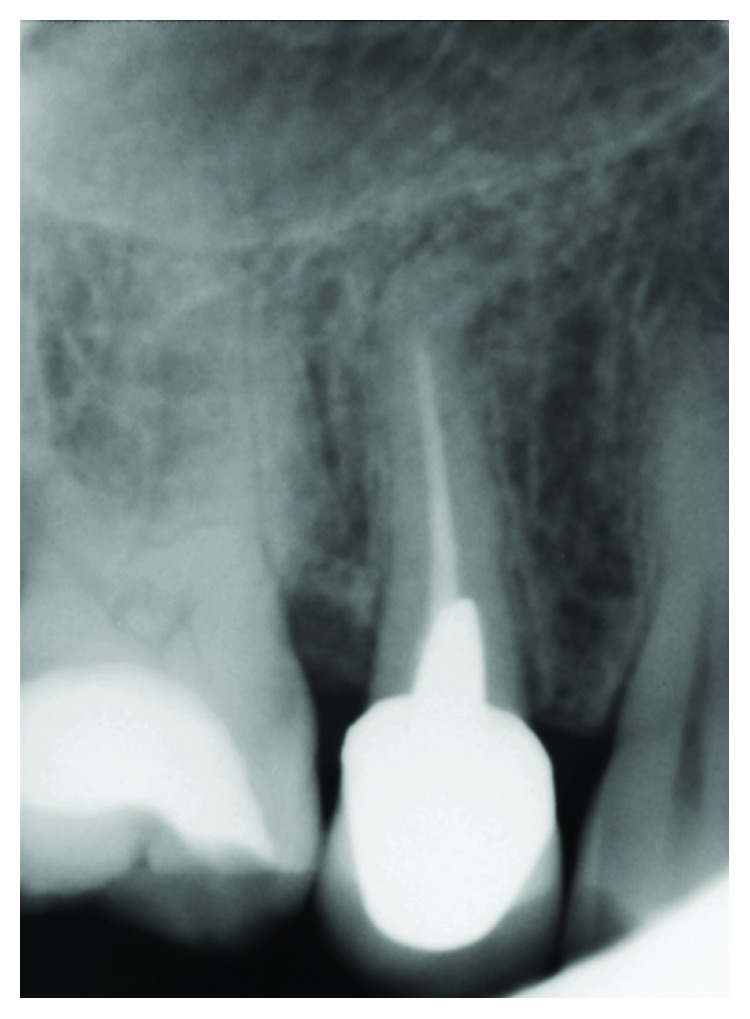
Periapical radiograph showing previous endodontic and prosthetic treatments on upper second premolar.

The previously treated abutment (>10 years ago, by other colleagues) was originally restored with a metal cast post-and-core extending up to the coronal third of the root; the intraoral periapical X-ray revealed a partially treated and/or filled root canal space, accompanied by a slight radiolucency at the mesial side of the apex (Figure 2). Periodontal clinical parameters were all within normal limits.

After a complete clinical and radiographic evaluation, preprosthetic treatments were deemed necessary for the second maxillary premolar: our efforts were addressed towards delivering an all-ceramic restoration, in order to satisfy high patient's expectations and esthetic needs.

### 2.3. Disassembly and Endodontic Retreatment

Previous metal-based reconstructions would have been replaced by resin-based materials, in association with the adoption of adhesive techniques.

**Figure 3:**
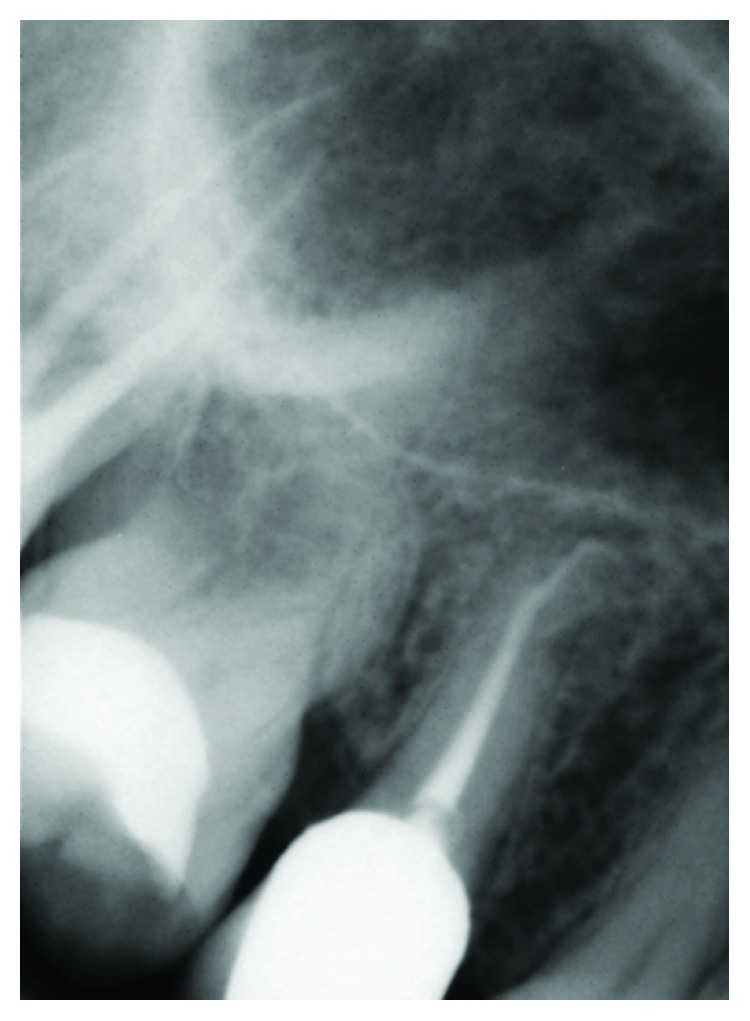
Periapical radiograph showing immediate outcome of nonsurgical endodontic retreatment performed at our dental office.

The preexisting crown was sectioned and gently removed. The abutment disassembly was accomplished with the aid of ultrasonic inserts until mobilization of cast post was obtained; then, a nonsurgical root canal retreatment (NSRCR) was planned. Standardized endodontic procedures included the use of stainless steel manual files and rotary Ni-Ti instruments (ProTaper® Universal Series, Dentsply Maillefer), along with NaOCl and EDTA irrigations; the single-cone gutta-percha technique was chosen for final obturation (Figure 3) of the root canal (finishing file instrument and gutta-percha point: size *F1*, ProTaper® Universal, Dentsply Maillefer).

### 2.4. Core Build-Up, Prosthetic Preparation, and Crown Delivery

The abutment was finally restored using a tapered translucent glass-fiber post (D.T. Light-Post®, Bisco Inc.) luted with dual-polymerizing resin cement (Clearfil® SA, Kuraray Medical Ltd.); core build-up was completed with universal nanohybrid composite (Clearfil Majesty™, Kuraray Medical Ltd.) applications. A full-crown prosthetic preparation with a chamfer finishing line was accomplished: axial (1.0 to 1.5 mm) and occlusal reductions (1.5 to 2.0 mm) were carried out according to all-ceramic restoration's guidelines [23]; the cervical margin width was approximately 0.8 mm.

Medium-grit followed by fine-grit diamond burs provided a smooth preparation; due to its high precision and physical performances [24], a VPS material was chosen for a one-step impression technique, in association with retraction cords for gingival displacement (Elite HD+ putty soft; Elite HD+ light body, Zhermack SpA, Badia Polesine, Italy).

**Figure 4:**
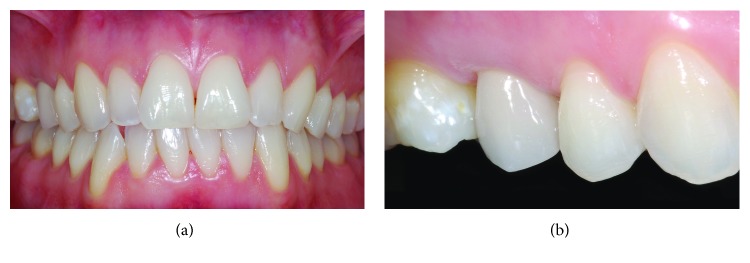
Postoperative intraoral frontal (a) and lateral (b) views showing good integration of the new veneered-zirconia crown on tooth 1.5.

The porcelain-zirconia restoration was CAD/CAM fabricated starting from a presintered zirconia blank (Zirkonzhan, GmbH), milled with a dedicated machine (5 + 1 axis milling unit, M5, Zirkonzhan GmbH); the framework was refined, completely sintered (Zirkonofen 600, Zirkonzahn GmbH), and veneered. The definitive crown was adhesively luted as previously reported [25]: briefly, the inner surface of zirconia framework was pretreated with low-pressure (1 bar) 50 *μ*m alumina sandblasting and ultrasonically cleaned; an MDP-based, dual polymerizing luting agent (Clearfil® SA, Kuraray Medical Ltd.) was subsequently applied for final cementation (Figures 4(a) and 4(b)). After luting, the occlusion was verified to avoid both interferences during excursive (protrusive and lateral) jaw movements and prematurities at maximum intercuspation.

### 2.5. Regular Check-Ups and Development of Symptoms

**Figure 5:**
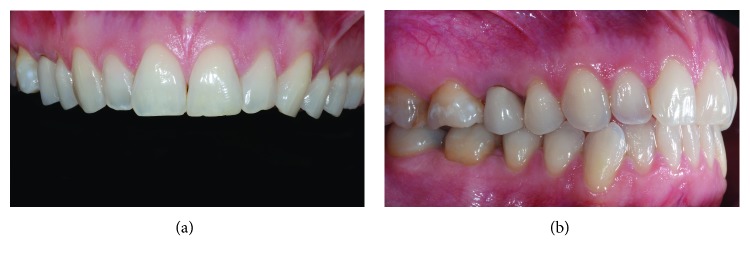
Frontal (a) and lateral (b) views 7 years after crown delivery: an overall dental deterioration is visible associated with soft tissue modifications.

The premolar rehabilitation was completed within the same year (2008), and the tooth entirely recovered its function in the mouth; regular check-ups (approximately every 6 months) were carried out during subsequent years. Marginal integrity, signs of wear and visible cracks of the artificial crown, shade matching, and development of secondary caries at the interface with the tooth were assessed during the check-ups [26, 27]: the clinical examinations included periodontal probing and were accompanied by radiographic analyses. While the patient received other dental therapies in the meantime, no further problems or complications related to the premolar treatment were detected at follow-ups. About seven years later, in 2015, some modifications of the soft tissues were noted, as multiple gingival recession developed on upper posterior teeth: however, the margin at the premolar restoration was just slightly affected (0.5 mm recession, Figures 5(a) and 5(b)). High compliance was shown by the patient throughout the treatment, as demonstrated by strict attendance at check-ups, adequate biofilm control, and copings with discontinuous pain/symptoms during later stages.

**Figure 6:**
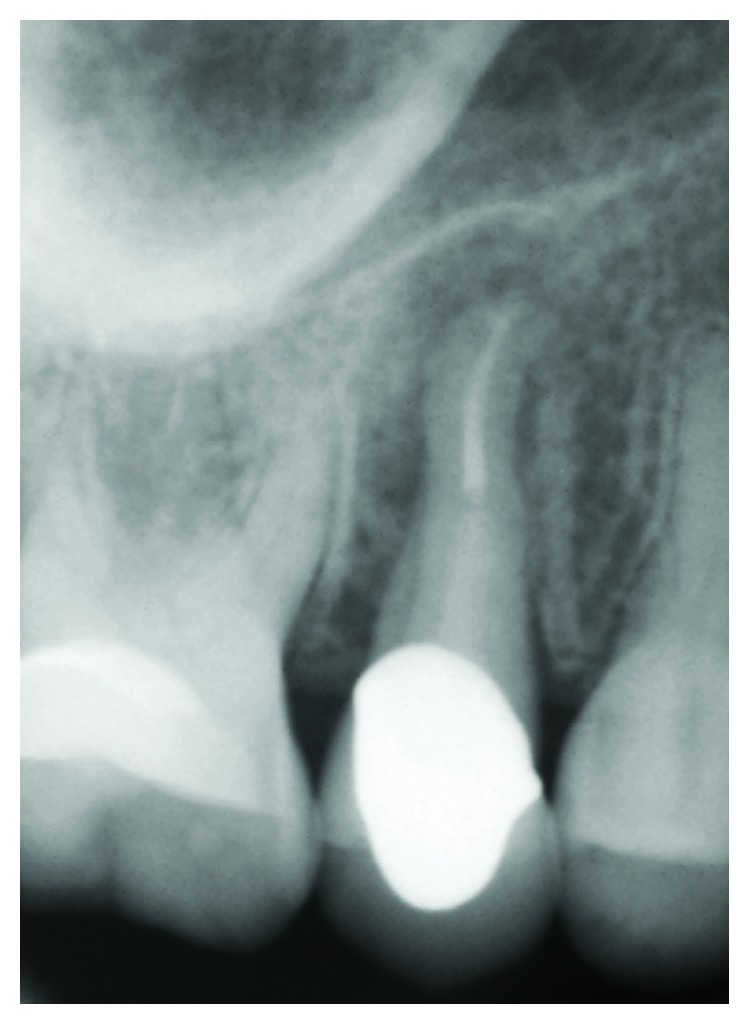
Development of clinical symptoms 9 years after crown delivery: the radiograph revealed a periapical radiolucency, with widening of periodontal ligament/lamina dura modifications.

Development of symptoms started about nine years later, in March 2017: at physical examination, a vestibular draining sinus tract on attached gingiva was discovered, with a mild positive response of the tooth to palpation (Miller class I score, for mobility test) and percussion tests. Diffuse widening of the periodontal ligament (in comparison with X-ray at time of root canal filling) and lamina dura modifications near the apex were observed from radiographic analysis, despite the apparently well-performed root canal therapy (Figure 6). At this time point, a deep vestibular pocket was also detected by the periodontal exam (manual probing depth > 10 mm). Due to clear alterations of the attachment system, the analyses of tooth mobility and pain on biting were also carried out at subsequent examinations. The occlusal status was checked, in order to identify potential trauma or overloads to the tooth: the patient presented a class I interarch relationship, with lateral canine guidance and absence of interferences during jaw movements. Minor signs of wear were identified at some locations (slight indentations at incisal margins of left central incisor, left lateral incisor, and canine: Figure 5(a)); the zirconia-porcelain crown, however, was free from chippings and visible cracks.

Following a pharmacologic treatment for acute phase management and resolution of the sinus tract, a surgical intervention was planned for several reasons: (1) direct inspection of potential fracture lines that were not visible on 2D radiographic images; (2) to assess the status of periradicular tissues and bone; and (3) to investigate the presence of accessory lateral canals, especially along the body (middle third) or at the apical mesial curvature of the root (last 3-4 mm). Patient and clinician's shared efforts were all addressed towards achieving a definite diagnosis and, possibly, tooth preservation: from this perspective, the open-flap surgical intervention was well accepted by the patient.

### 2.6. Surgical Procedure

**Figure 7:**
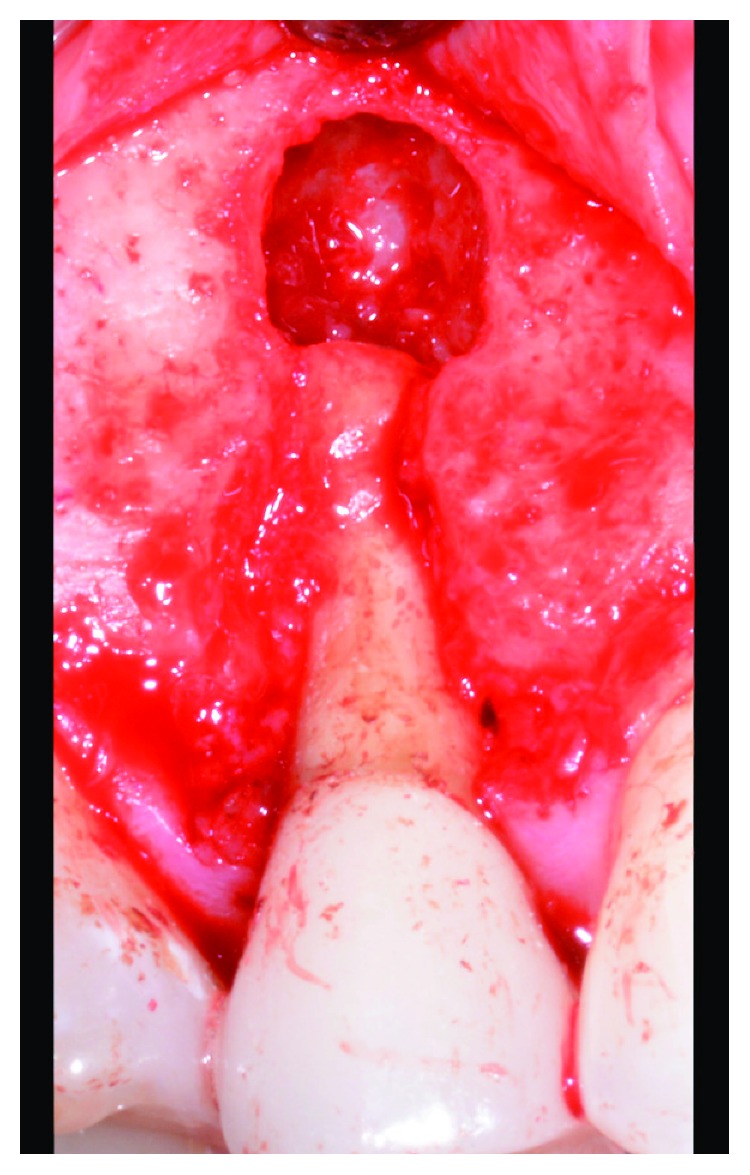
Apical resection of premolar root showing vertical bone resorption at the cortical vestibular side.

During May 2017, a papilla-sparing, trapezoidal, full-thickness flap was reflected and extensive cortical bone loss was confirmed at the vestibular side of the root. Under magnification and fiber-optic illumination, however, the exposed area of the root appeared free of cracking lines. Interproximal bone peaks were still preserved. A root-end resection was performed to ensure the removal of apical ramifications and/or residual bacterial contamination as potential aetiologic factors (Figure 7). The periapical granulation tissue was carefully removed with the aid of curettes and hand excavators; the residual cleaned cavity was finally irrigated with saline solution. The flap was repositioned and sutured.

**Figure 8:**
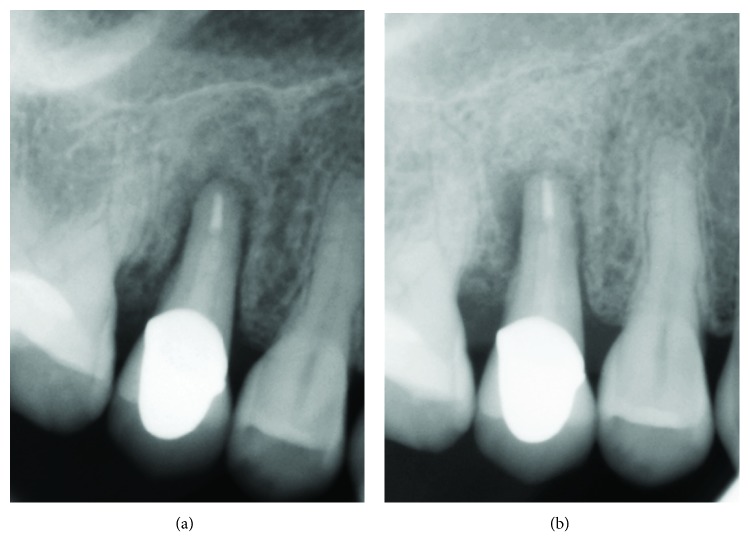
Three (a) and five (b) months after endodontic surgery, the clinical scenario did not improve: a progressive radiolucency involved the distal areas of the tooth and proximal peaks.

The clinical scenario did not improve after apical surgery: during subsequent months (July and September 2017), the radiographic follow-up revealed a progressive radiolucency also involving the distal areas of the tooth and proximal peaks (Figures 8(a) and 8(b)). Increased horizontal tooth mobility (Miller class II score) and pain on biting at the premolar tooth were present in September 2017.

### 2.7. Vertical Root Fracture

**Figure 9:**
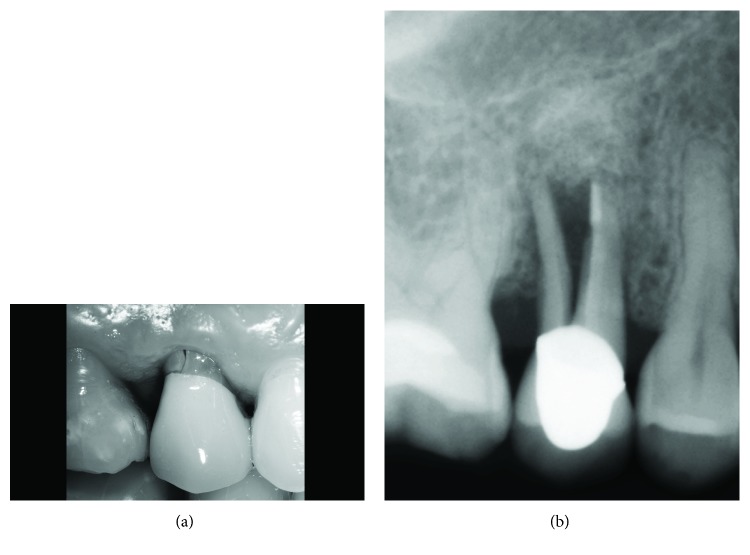
Clinical (a) and radiological (b) presentation of the vertical root fracture: splitting of the premolar root into two halves is clearly visible.

**Figure 10:**
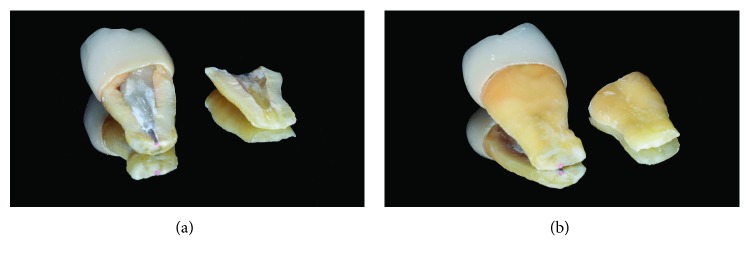
Inner (a) and outer (b) views of the two fragments produced by the longitudinal fracture, retrieved after tooth extraction.

**Figure 11:**
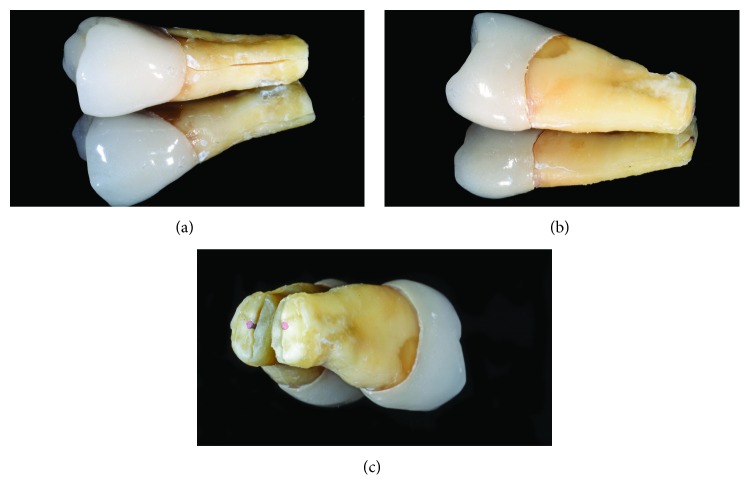
Extraoral close readaptation of fragments: the fracture runs for the entire bucco-lingual length of the tooth, as shown by separation on the vestibular (a) and palatal (b) sides. The fracture line was off-centered from the canal when observed on the axial plane (c).

**Figure 12:**
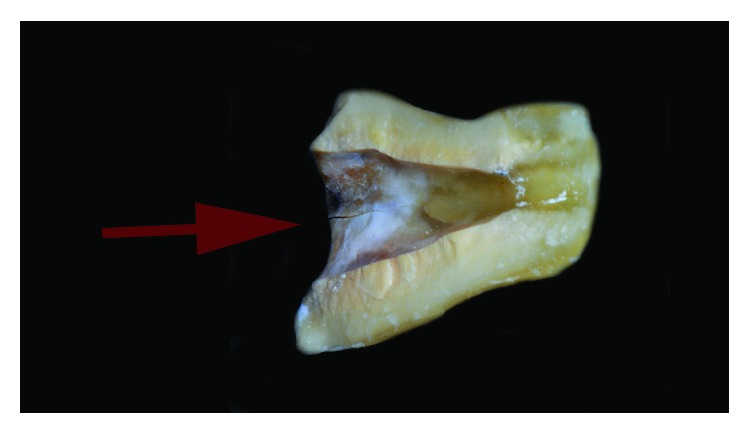
An incomplete cracking line (arrow) involved the coronal area of the small fragment.

On December 2017, a vertical fracture associated with separation of root fragments came to light both clinically and radiographically (Figures 9(a) and 9(b)). The vestibular view showed a clear, 1 mm wide gap between the two root halves, running up to gingival margin of the prosthetic crown. Considering the extensive periodontal breakdown, type of fracture, and apical splitting, the second upper premolar was scheduled for extraction. Despite some emerging treatments are available for VRF, like fragment reattachment and tooth replantation, they still need long-term validation [28]; in order to prevent further bone loss and achieve rapid resolution of symptoms, the extraction treatment was proposed and accepted by the patient. Two main tooth fragments were retrieved from the extraction procedure: a larger one, formed by the crown, post and gutta-percha obturation and a smaller slice with unoccupied root canal space, detached from all the other restorative materials (Figures 10(a) and 10(b)). The fracture was running for the entire bucco-lingual root length: it was considered “complete” in the horizontal extent or type “A” according to the classification of von Arx and Bosshardt [16], being visible from both the vestibular and palatal sides (Figures 11(a) and 11(b)). On the longitudinal plane, the cracking line was also complete, extending from the prosthetic margin to the root-end resection. The fracture was off-centered in the axial plane (i.e., asymmetric involvement of the root), locating itself mainly outside the root canal space in the apical third (Figure 11(c)). A detailed analysis of the fragments shows a close adaptation between post/endodontic filling and the dentinal walls, which also appeared of adequate thickness; in addition, an incomplete cracking line was visible on the coronal third of the small fragment (Figure 12).

## 3. Discussion

### 3.1. Type of Complications for Single-Tooth Restorations

Biological and technical complications are currently reported in the literature for zirconia-based, tooth-supported single crowns. The predominant recorded failures during the first 5–7 years of service were technical, related to the prosthesis: according to Monaco et al. [26], delamination of the veneering ceramic, also known as chipping, was frequently associated with parafunctional habits of the patients. Rinke et al. also reported fractures of the veneering material (12.4% of the considered crowns, observation time: 7 years), along with crown decementations (10% of the considered crowns) [15]. That kind of technical complications might require a minimally invasive intervention. In fact, loss of retention is usually managed with adhesive reluting; polishing/composite repair or crown replacement might be selected for minor and major chipping, respectively. In our study, the premolar crown was not affected by any technical problems during the entire study period: the postextraction analysis also showed optimal marginal accuracy and fitting of the restoration. In other words, survival and success (no occurrence of postcementation complications, up to the extraction procedure) related exclusively to the restoration itself were demonstrated.

Biological failures, on the other hand, are less frequently encountered and strictly related to the supporting tooth: they include secondary caries, periodontal disease, or structural problems such as fractures [15].

In our study, the occurrence of a complete VRF was relatively unexpected in relation to the time elapsed from initial operative procedures (about 9 years). In fact, according to the study of Fuss et al. [22], 50% of the extractions due to VRF were recorded between 1 year and 5 years after root canal treatment or retreatment, while 18% of teeth failed within 1 year from endodontic procedures. Pradeep Kumar et al. also reported that pulpless teeth covered with crowns are more likely to develop VRFs within 5 years postoperatively (mean time of 4.35 ± 1.95 years) [19]. Among restored teeth, premolars could be particularly affected by fractures due to anatomical reasons: (1) their crowns are bulkier than anterior teeth (incisors and canines) but they show reduced mesio-distal diameters of the roots: second maxillary premolars, in addition, are usually single-rooted; (2) premolars are characterized by crowns with steep cuspal inclination and are located midway (between molars and anteriors) along the occlusal arch: in this way, they are subjected to significant lateral forces during functional and parafunctional activities. Ferrari et al. [29] carried out a cornerstone randomized controlled trial on endodontically treated and restored/crowned premolars, showing the importance of coronal tissue preservation: they found the highest number of root fractures on compromised teeth with one or two residual coronal walls; on the other hand, no failures (cracks) were recorded on elements with 3 and 4 preserved walls.

### 3.2. Aetiological Factors Related to VRF

Recovery of compromised teeth might include a number of preprosthetic steps that have been identified as risk factors for development of VRF: from a chronological perspective, aetiological variables usually described in the literature play a role before the placement of a full-occlusal coverage. The impact of endodontic procedures on structural integrity of teeth and retreatments, in particular, have been investigated [19, 21, 22]: canal shaping appears to cause stresses in dentin along with apical microcracks, regardless the type of rotary instrument motion [30, 31]; further propagation and extension of that partial fractures might be sustained by functional occlusal loads, despite the existence of a full-crown restoration. The use of chemical agents (i.e., irrigants) has been associated with a deterioration of dentinal properties of nonvital teeth [32, 33]. With endodontic retreatments, both mechanical and chemical agents are applied once again on inner root surfaces, producing a relative enlargement of the canal walls and increased loss of radicular dentin: in fact, endodontically retreated teeth have shown a reduced resistance to fracture when compared to first-time treated elements [34]. According to the above data, we may speculate that microcrack development or propagation, in our study, may have been facilitated by the endodontic retreatment procedures and/or by the removal of preexisting cast post. Among filling techniques, however, the adopted single-cone obturation is considered relatively safe regarding the development of lateral condensation forces [30].

An increased risk of fractures could be associated with repetitive restorative cycles: they lead to a progressive removal of dental tissues and should be avoided [35]. During a patient's life, multiple replacements of artificial crowns may be required for a wide number of reasons: aging and wear, loss of marginal integrity, fractures, caries at the interface, and endodontic problems [36]. In addition, previous indirect restorations might become damaged, inaccurate, and in need of replacement when performing disassembly and/or adequate access for root canal retreatments. In our case, a new restorative cycle was started mainly for esthetic and endodontic reasons. Delivering state-of-the-art first-time treatments along with adoption of durable prosthetic materials might reduce the need for new restorative cycles [35].

### 3.3. Diagnostic Challenges

Despite technological improvements, a clear detection of VRFs is still a clinical challenge: early signs are similar to those of other conditions such as periodontal disease, apical periodontitis, or combined endo-perio lesions [21, 37]. According to the results of Yoshino et al. [37], a definite diagnosis of longitudinal fracture was established about 18 months after the initial onset of clinical symptoms, on upper second premolars; diagnostic rate for VRF was just nearly 50% at 12 months and 79.5% at 24 months.

In the present study, the time span from initial symptoms to final diagnosis was 9 months (from March to December 2017): no dislodgment of the crown/build-up restoration and absence of cracking lines during first months were the main misguiding factors. On the other hand, mild symptoms, positive periodontal probing, and progressive enlargement of lamina dura (radiolucent halo) were typical features of root fracture's presentation [21, 38]. The challenges related to identification of a VRF were also explained to the patient: after proper communication, she was confident and allowed the clinician adequate time for follow-ups and reevaluations, in order to reach a final diagnosis.

While a CBCT exam was not performed, the diagnostic capabilities of that radiological instrument have not been fully explored: the in vivo accuracy of fracture detection, for crack width in the range of 50–330 *μ*m, was low [39]; in addition, filling materials and posts in the canal may also impair clear visualization of VRF.

### 3.4. Limitations of the Study and Future Directions

A careful evaluation and control of patient-based or tooth-related variables, along with specific identification of VRF's origin, are not possible with a case report study design. The description of our clinical event, on the other hand, might be helpful to understand features, timing, and presentation's mode of a serious long-term complication. The mechanical behavior of the tooth-restoration unit, when high-strength ceramic frameworks are chosen, should be further explored. In particular, future research should be performed to evaluate force transmission or dissipation from all-ceramic coronal restorations to the roots of endodontically treated teeth.

### 3.5. Final Remarks

Potential long-term complications should be taken into account when dealing with previously treated and/or structurally weakened teeth. The development of a vertical root fracture might be unexpected and might occur many years after the end of a tooth rehabilitation.

Patients should always be aware and well informed about risks associated with recovery of compromised teeth and their prosthetic rehabilitations.
